# KinetochoreDB: a comprehensive online resource for the kinetochore and its related proteins

**DOI:** 10.1093/database/baw019

**Published:** 2016-03-17

**Authors:** Chen Li, Steve Androulakis, Ashley M. Buckle, Jiangning Song

**Affiliations:** 1 Infection and Immunity Program, Biomedicine Discovery Institute and the Department of Biochemistry and Molecular Biology, Faculty of Medicine, Monash University, Melbourne, Victoria, 3800, Australia; 2 Monash Bioinformatics Platform, Monash University, Melbourne, Victoria, 3800, Australia; 3 Monash Centre for Data Science, Faculty of Information Technology, Monash University, Victoria, 3800, Australia, and; 4 National Engineering Laboratory of Industrial Enzymes and Key Laboratory of Systems Microbial Biotechnology, Tianjin Institute of Industrial Biotechnology, Chinese Academy of Sciences, Tianjin, 300308, China

## Abstract

KinetochoreDB is an online resource for the kinetochore and its related proteins. It provides comprehensive annotations on 1554 related protein entries in terms of their amino acid sequence, protein domain context, protein 3D structure, predicted intrinsically disordered region, protein–protein interaction, post-translational modification site, functional domain and key metabolic/signaling pathways, integrating several public databases, computational annotations and experimental results. KinetochoreDB provides interactive and customizable search and data display functions that allow users to interrogate the database in an efficient and user-friendly manner. It uses PSI-BLAST searches to retrieve the homologs of all entries and generate multiple sequence alignments that contain important evolutionary information. This knowledgebase also provides annotations of single point mutations for entries with respect to their pathogenicity, which may be useful for generation of new hypotheses on their functions, as well as follow-up studies of human diseases.

**Database URL:** http://lightning.med.monash.edu/kinetochoreDB2/

## Introduction

During cell mitosis and meiosis, the kinetochore plays a critical role of locating the attachments on chromosomes and pulling sister chromatids apart. Assembled on centromeric chromatin, kinetochore functions during the cell cycle (1–6). During the last few decades, numerous studies of the kinetochore and its related proteins have characterized its function, architecture and the repertoire of its related proteins using biochemistry, structural biology and cell biology techniques (4, 7–11). Both the stability of the kinetochore–microtubule interface and mutations occurring in the kinetochore and its related proteins are associated with a number of human diseases (12–15). Dynamics studies of the kinetochore have also shown that deregulation of the kinetochore-microtubule dynamics frequently results in chromosome instability, leading to the development of cancer (10, 11, 16). Other experimental studies reveal that mutations of the kinetochore and its related proteins are closely linked to human diseases. For example, the adenomatous polyposis coli protein, localized in both centrosome and kinetochore, contains ∼15 disease-associated mutations that cause familial adenomatous polyposis (14, 15) and Medulloblastoma (12).

Despite its biological significance and our increasing awareness of its potential roles in human diseases, there is currently a paucity of publically available databases or resources that focus on providing comprehensive functional annotations of the kinetochore and its related proteins. The only available database is MiCroKiTS (17), an integrated online resource for kinetochore, midbody, telomere, centrosome and spindle proteins. However, important annotations of entries in MiCroKiTS are not available in terms of protein 3D structure, protein interaction partners, metabolic/signaling pathways etc., all of which are crucial aspects for follow-up functional studies of these proteins.

In an effort to address this knowledge gap, we created KinetochoreDB, which integrates several public databases, computational annotations and experimental results for currently 1554 related entries. KinetochoreDB contains several important features, the majority of which are not available in MiCroKiTS (Table 1):

**Table 1.** Comparison between KinetochoreDB and MiCroKiTS

**Annotation category**	**KinetochoreDB**	**MiCroKiTS**
Target	Kinetochore and its related proteins	Kinetochore, centrosome, midbody, telomere and spindle proteins
Biological model	Yes	No
Protein 3D structure	Yes, detailed structural information available; customizable display	No
Protein intrinsic disorder	Yes, predicted by VSL2B	No
Protein interaction partner	Yes, detailed information available	No
Metabolic/signaling pathway	Yes	No
Disease-associated mutations	Yes, incorporating both disease-associated and nonsense mutations	No
Evolutionary conservation	Yes, MSAs curated and visualized using Jalview	No MSA
User enquiry and submission	Yes	No

- It provides annotations of protein 3D structure when structural information is available. For protein entries with available structural information, the corresponding Protein Data Bank (PDB) IDs and their related information are provided. In addition, information on predicted intrinsic disorder is provided, which is particularly important for providing structural insights into those entries in KinetochoreDB whose 3D structures have not been solved.
- It provides comprehensive annotations of single point mutations and their pathogenic effects. These mutations are classified as either pathogenic or nonsense mutations in KinetochoreDB. For disease-associated pathogenic mutations, KinetochoreDB provides details of the disease caused by the mutation, allows users to search the entire database with the disease name of interest, and provides user-friendly options to browse the related kinetochore proteins that harbor such disease-associated mutations.
- It provides metabolic/signaling pathway information for each entry by cross-referencing the KEGG database. Such information is important for understanding the functions of kinetochore proteins from a biochemical network perspective. In particular, the pathway information and the link to Kyoto Encyclopedia of Genes and Genomes (KEGG) will be provided if an entry has pathway information available in KEGG.
- It provides multiple sequence alignments (MSAs) for all included entries, thereby allowing users to readily identify evolutionarily conserved regions within the family of a kinetochore protein. In addition, the visualization of MSAs implemented by Jalview is user-friendly and customizable.
- It provides convenient user enquiry and new entry submission options by enabling users to automatically upload their newly discovered sequences into the online database.

## Materials and Methods for Database Construction

**Figure 1.**
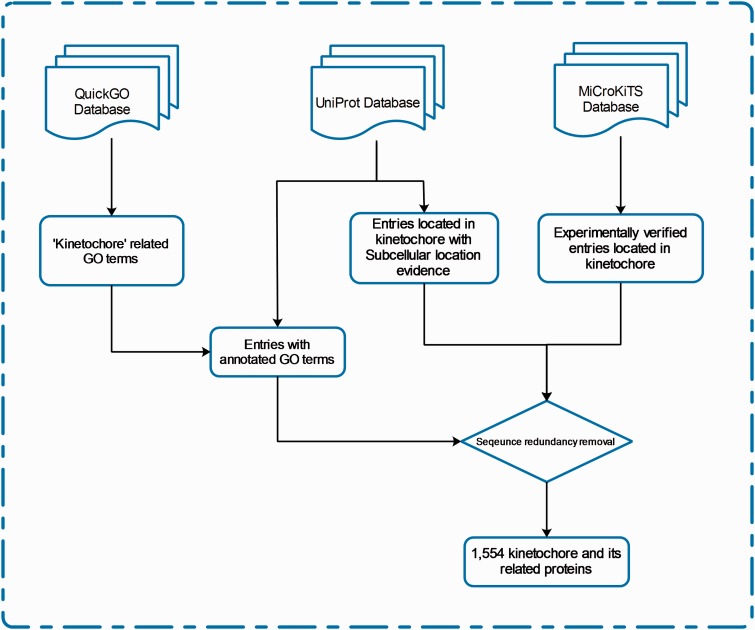
The schema of database construction and data collection processes.

**Figure 2.**
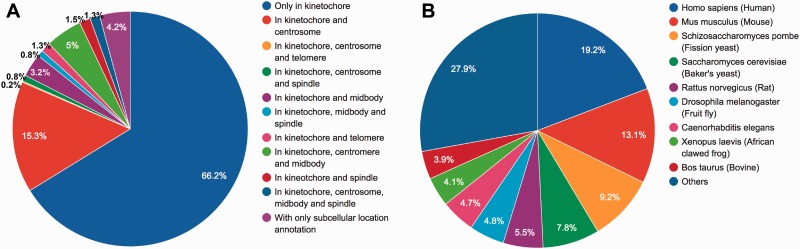
Statistical summary of locations and species of KinetochoreDB entries. **(A)** Distribution of protein locations according to MiCroKiTS and protein subcellular location annotations from UniProt (entries with GO annotations only were excluded). **(B)** Distribution of species of all KinetochoreDB entries.

We define ‘kinetochore and its related proteins’ with respect to protein subcellular location and Gene Ontology (GO). The entries of KinetochoreDB originate from three major resources; QuickGo database (18), UniProt database (19) and MiCroKiTS, and the database was populated as follows. From MiCroKiTS, we obtained data entries that have been experimentally verified to be located in kinetochore. By searching GO terms from QuickGo with the keyword ‘kinetochore’, we obtained 64 GO terms related to kinetochore. For each GO term, we searched and filtered the reviewed entries from the UniProt database to ensure that all the downloaded entries contain the GO annotation. Applying this procedure resulted in 53 GO terms including 25 cellular component terms, 2 molecular function terms and 26 biological process terms (Supplementary Table S1). In addition, we queried ‘subcellular location’ with the keyword ‘kinetochore’ in UniProt and downloaded the entries with published experimental evidence from the search results. After the removal of redundant entries, the resulting dataset contained a total of 1554 carefully reviewed entries. The detailed procedures of database construction and data collection are illustrated in Figure 1 and a statistical summary can be found in Figure 2 and Table 2, respectively.

**Table 2.** Statistical summary of the information contained in KinetochoreDB

Number of entries	1554
Number of protein structures	1232
Number of protein interactions	49 931
Number of biological models	55
Number of mutations (including reside substitution, insertion and deletion)	1424
Number of KEGG pathways	202
Number of Pfam domains	1173
Number of PTMs sites	4027

For each entry, KinetochoreDB integrates several public resources, including the UniProt database, NCBI Gene database (http://www.ncbi.nlm.nih.gov/gene/), 1000 Genome Project database (20) (for human proteins), BioModels database (21), Research Collaboratory for Structural Bioinformatics (RCSB) PDB (22), Online Mendelian Inheritance in Man (23), BioGRID 3.2 (24), Pfam database (25) and KEGG (26), in order to provide a comprehensive description with respect to basic protein information, protein structure, function, mutation and evolutionary conservation. An important feature of KinetochoreDB is the provision of 3D structure. To achieve this, we manually searched all the entries against the PDB database using their corresponding UniProt identifiers and protein names. For protein complex structures, we identified the PDB chain for each entry and annotated the entry with that chain. In addition, we also generated MSAs using all homologous sequences for each protein entry. Homologous sequences were retrieved by PSI-BLAST (27) search against the Swiss-Prot dataset obtained from UniProt. The alignments were generated using Clustal Omega (28). We also predicted natively disordered regions for all protein entries using one of the most widely used disorder predictors, namely VSL2B (29). A residue is annotated as disordered by VSL2B if its prediction score was >0.5.

We used Jmol (http://jmol.sourceforge.net/) and pViz (30) for visualization of protein structures, and Jalview (31) for customizable editing and display of MSAs for each protein entry. In order to provide the annotations of the domain context including functional domains and sites for each protein entry in KinetochoreDB, another Javascript based plug-in, The Protein Feature View (http://andreasprlic.github.io/proteinfeatureview/), was used to display protein structural, functional domains and sites in a better and more interactive way. The display of protein domain context can be found in ‘Protein Domain Context’ section of the webpage. The information stored in KinetochoreDB resides in a MySQL relational database. A highly interactive web front-end to the data was implemented using the Javascript framework, JQuery. Apache Tomcat handles serving of data to users on the web, utilizing a set of Java Servlets and JavaServer Pages for data searching and viewing.

## Database Utility

**Figure 3.**
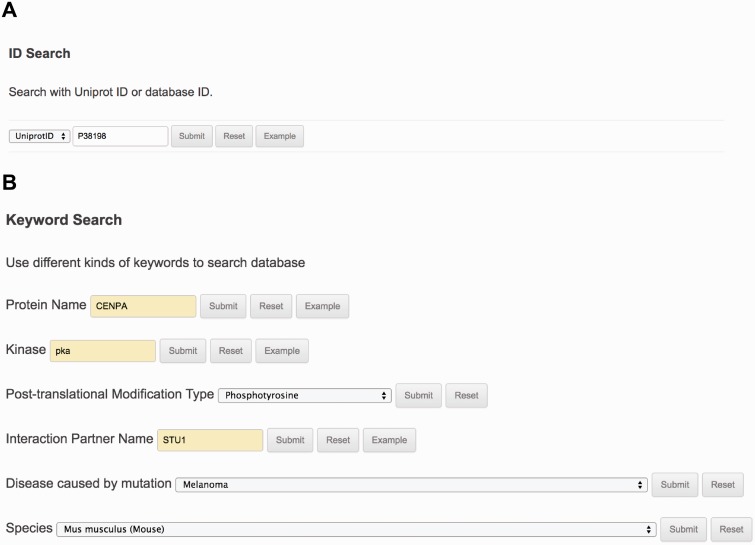
The search interface and options provided by KinetochoreDB. **(A)** Protein ID search with either UniProt ID or KinetochoreDB ID. **(B)** Keyword search with the protein name, kinase, PTM type, interaction partner name, disease or species.

**Figure 4.**
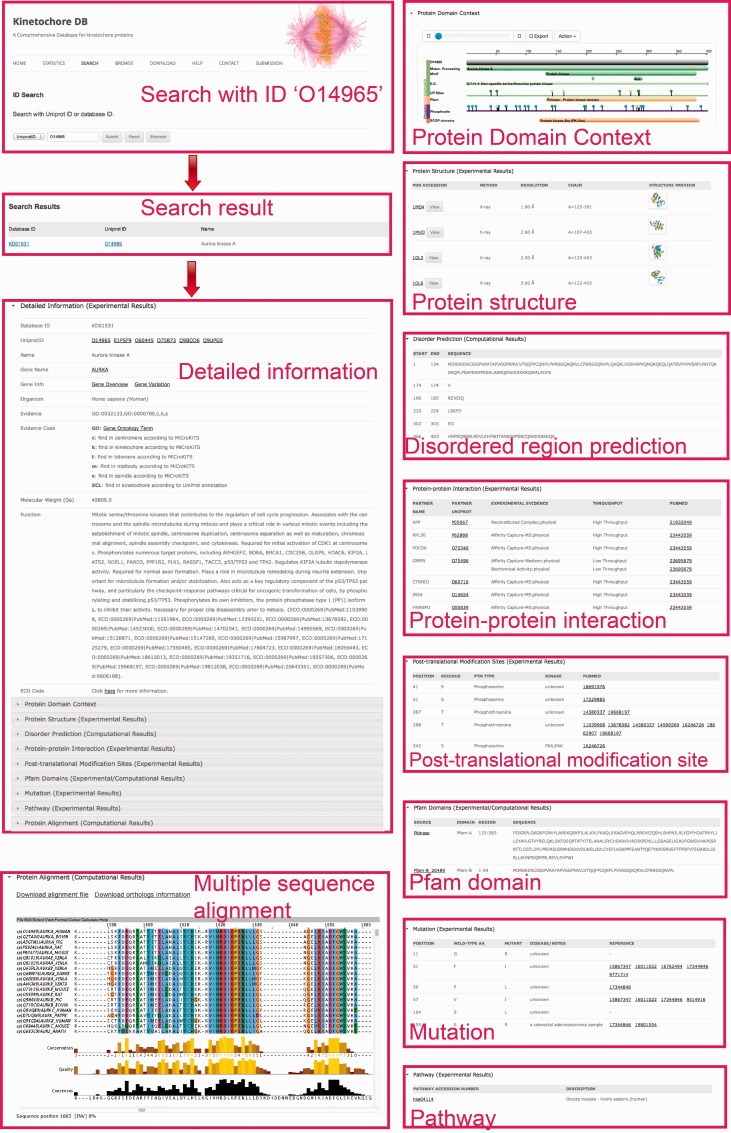
A webpage showing protein detailed information for search results in KinetochoreDB using the UniProt ID O14965 as the query. The results are classified and organized in ten sections including protein information, protein domain context, protein structure, disorder region prediction, PTMs, protein interaction, protein mutation, Pfam domain, metabolic/signaling pathway and MSA.

The ‘Search’ page (http://lightning.med.monash.edu/kinetochoreDB2/Search.jsp) (Figure 3) allows users to search the database in several different ways. These search options can be generally classified into two groups: search with ID or search with keywords. Examples are provided below to assist users to understand how to perform the search. When searching the database with IDs, KinetochoreDB provides two different kinds of IDs to facilitate the search: UniProt ID and KinetochoreDB ID. The latter is composed of ‘KD’ and five digits, e.g. KD00095. Considering that the database includes 1554 entries in total, each entry is accordingly numbered as KD00001–KD01554. In addition KinetochoreDB offers alternative search options with keywords. These include protein name, kinase name, post-translational modification (PTM) type, name of protein interaction partner and name of diseases caused by single point mutations (Figure 3B). After selecting the ‘Submit’ button, the corresponding search results will be shown on the webpage. For each entry, there are generally 10 sections of structural and functional categories, including general information, protein domain context, protein structure, disordered regions prediction, interaction partner, PTMs, Pfam domain, protein mutation, metabolic/signaling pathway and protein alignment with homologs. To provide an illustration of the annotations for each entry in KinetochoreDB, we use ‘UniProt ID = O14965’ (KinetochoreDB ID = ‘KD01531’) as an example query. The resulting page with ten sections is shown in Figure 4.

For protein overview and domain context, KinetochoreDB uses The Protein Feature View plug-in to facilitate the general description of protein entries including functional sites and domains (Supplementary Figure S1A), allowing a more detailed inspection of the constituent domains of the protein. For protein structure overview, KinetochoreDB provides two different ways to inspect the 3D structures. A single structure for the current protein entry can be examined by clicking the ‘View’ button to launch Jmol, a Java applet for displaying 3D structures (Supplementary Figure S1C). Multiple structures can also be viewed together as an ensemble using pViz (Supplementary Figure S1B).

With respect to protein–protein interaction, the interaction partner is highlighted if this protein is also an entry of KinetochoreDB. As a result of our search strategy (see ‘Materials and Methods for Database Construction’ for details), certain proteins in MSAs might not be included in the current KinetochoreDB. To facilitate the comparison between entries in KinetochoreDB and their homologs, we archived the homologs by extracting protein UniProt IDs. Detailed information for these files is available in the ‘Protein alignment’ section of the webpage.

KinetochoreDB will be updated on a regular basis (typically every 2–3 months) to include newly available entries from various resources, in order to allow an up-to-date archive of recent results of the kinetochore and its related proteins. All the updates will be carefully reviewed prior to their release. In addition, we also allow users to submit their research findings related to kinetochore to our KinetochoreDB. New submission should include protein/gene detailed information (such as sequence, molecular weight and protein function description), protein structural information, protein functional annotation, single point mutation and its pathogenicity and metabolic/signaling pathway (Supplementary Figure S2). After careful review and validation, new data will be included in KinetochoreDB and made publically available.

## Discussion

**Figure 5.**
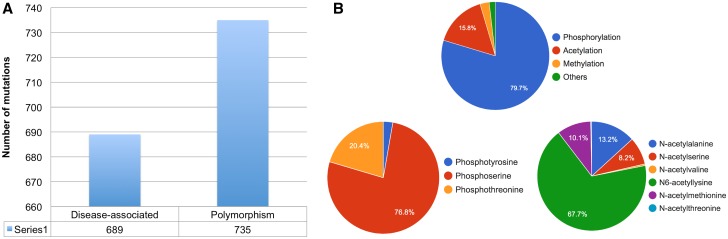
Statistical analysis of single point mutations and protein PTM types in KinetochoreDB. **(A)** Distribution of disease-associated mutations and polymorphisms. **(B)** Distribution of different major types of protein PTM, e.g. phosphorylation, acetylation, methylation and others. The distribution of sub-types of phosphorylation and acetylation is also shown.

**Figure 6.**
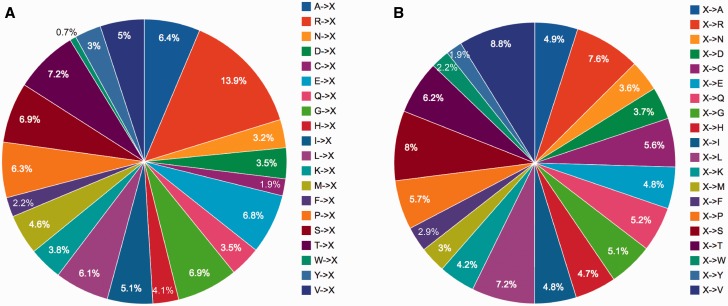
Statistical analysis of single point mutations in KinetochoreDB according to two mutation patterns (**A**) (A…V)->X and (**B**) X-> (A…V), where X denote any type of amino acid).

Certain protein entries in KinetochoreDB harbor mutations. We thus provide a brief statistical analysis of these mutations. A total of 1424 mutations were found to occur in the 206 entries in KinetochoreDB. Among these, 689 (48.4%) mutations were found to cause diseases, while 735 (51.6%) were nonsense mutations (Figure 5A). PTMs, on the other hand, extend the chemical repertoire of amino acids by attaching new chemical groups and small molecules to the side chains of amino acids. Using the available PTM annotations in KinetochoreDB, we analysed the distribution of different types of PTMs for all the entries. We note that the kinetochore and its related proteins possess many PTM sites, the top three of which are phosphorylation, acetylation and methylation, respectively (Figure 5B). The distribution of different subtypes of acetylation and phosphorylation is also shown in Figure 5B. We further plotted the distributions of different types of mutations in Figure 6. We can see that there is no apprarent difference between the two types of mutation patterns (Figure 6).

**Figure 7.**
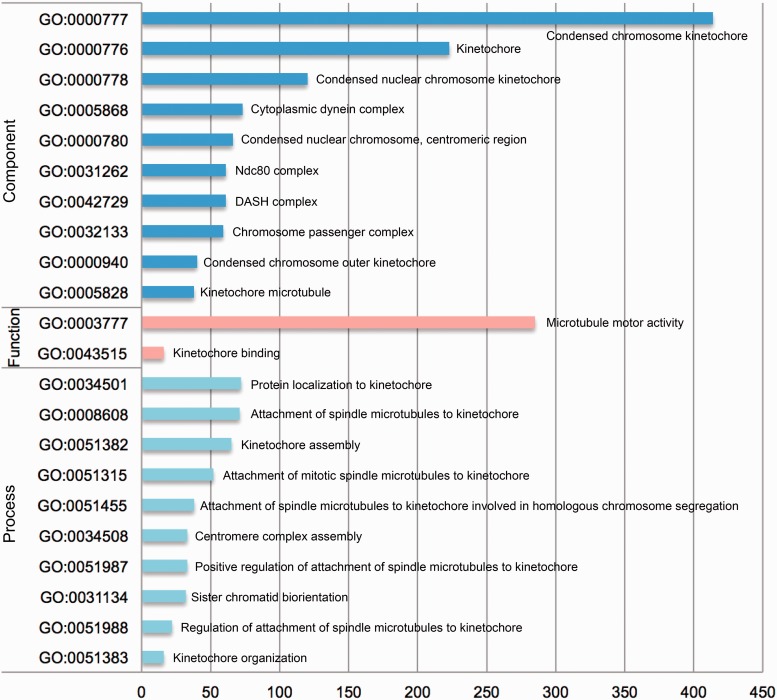
Statistical analysis of the number of entries from KinetochoreDB involved in different GO terms, which were grouped according to three categories: cellular component, molecular function and cellular process.

In addition, with the comprehensive dataset from KinetochoreDB, we conducted a statistical analysis of the number of proteins involved in different GO terms including cellular component, molecular function and biological process. The results are shown in Figure 7. For cellular component, the 10 top ranked GO terms are condensed chromosome kinetochore (GO:0000777), kinetochore (GO:0000776), condensed nucler choromosome kinetochore (GO:0000778), cytoplasmic dynein complex (GO:0005868), condensed nuclear chromosome, centromeric region (GO:0000780), Ndc80 complex (GO:0031262), DASH complex (GO:0042729), Chromosome passenger complex (GO:0032133), Condensed chromosome outer kinetochore (GO:0000940) and kinetochore microtubule (GO:00005828). For molecular functioin, the top ranked GO terms are microtube motor activity (GO:0003777) and kinetochore binding (GO:0043515). For biological process, the 10 top ranked GO terms are protein localization to kinetochore (GO:0034501), attachment of spindle microtubules to kinetochore (GO:0008608), kinetochore assembly (GO:0051382), attachment of mitotic spindle microtubules to kinetochore (GO:0051315), attachment of spindle microtubules to kinetochore involved in homologous chromsome segregation (GO:0051455), centromere complexe assembly (GO:0034508), positive regulation of attachment of spindle microtubules to kinetochore (GO:0051987), sister chromatid biorientation (GO:0031134), regulation of attachment of spindle microtubules to kinetochore (GO:0051988) and kinetochore organization (GO:0051383). More specifically, 414, 285 and 72 proteins contain the annotation of condensed chromosome kinetochore (GO:0000777), microtubule motor activity (GO:0003777) and protein localization to kinetochore (GO:0034501).

It should be noted that the statistical results regarding the PTM sites, mutations and GO terms are merely observations based on the entries in KinetochoreDB, rather than being interpreted as a biologically siginificant finding, as the entries included in KinetochoreDB only represent a subset of the entire proteome and are from different species.

## Conclusions

The kinetochore and its related proteins play extremely important roles during cell division and mitosis. In the past few decades, research on this topic has attracted a great deal of interest, not only because they are important for the cell cycle, mitosis and meiosis (1–6), but also because they harbor mutations that can cause human diseases (12–15). In this context, databases such as KinetochoreDB that provide comprehensive annotations on the repertoire of kinetochore-related proteins will greatly facilitate in-depth functional investigation of these proteins and their relationships with human diseases. Through effective data integration from multiple public resources, KinetochoreDB has collected large amounts of information for related protein entries with respect to their amino acid sequence, protein 3D structure, biological function and evolutionary conservation. By providing comprehensive functional annotations of all available kinetochore-related proteins, we believe that this online resource will be used as a powerful tool to bridge functional characterization and disease-associated mutation studies of this important class of proteins.

In the future, we will endeavor to improve and update the annotations and analysis of data entries in KinetochoreDB by the following means: (i) We will keep the database updated and provide up-to-date information to synchronize with the research progress in the kinetochore and its related proteins; (ii) We will integrate genomic information into our database and source these data from publicly available information or bioinformatics programs. These include coding sequence, transcription factor binding site, enhancer, promoter and other upstream or downstream regulatory information; (iii) We will combine other state-of-the-art predictors to annotate the natively disordered regions of all entries in the database, while highlighting the consensus prediction. Meanwhile, we will also collect experimentally verified disordered regions from DisProt (32), the most comprehensive resource dedicated to annotating disordered region of proteins; (iv) we will encourage experimental biologists to contribute to the development of KinetochoreDB by submitting their recent findings by making available newly added entries in the database after careful review.

In addition we will continue to improve and update the annotations and analysis of all entries in KinetochoreDB by implementing secondary analysis functions of the database and by integrating high-throughput experimental data. In particular, we will explore gene expression microarray data, transcriptomics and proteomics and detailed functional pathway data, so as to provide a comprehensive useful resource for the wider research community.

## Supplementary data

Supplementary data are available at Database Online.

Supplementary Data
