# Supplementary material for: KinetochoreDB: a comprehensive online resource for the kinetochore and its related proteins
Source: Database (Oxford). 2016 Mar 17;2016:baw019. doi: 10.1093/database/baw019 (PMC4795933; doi:10.1093/database/baw019)
Supplement: Supplementary Data [file supp_2016_baw019_index.html]

Supplementary Data 

# KinetochoreDB: a comprehensive online resource for the kinetochore and its related proteins

## Supplementary Data

files

- Supplementary Data - docx file
