# Supplementary material for: KinetochoreDB: a comprehensive online resource for the kinetochore and its related proteins
Source: Database (Oxford). 2016 Mar 17;2016:baw019. doi: 10.1093/database/baw019 (PMC4795933; doi:10.1093/database/baw019)
Supplement: Supplementary Data [file supp_baw019_KinetochoreDBSuppRevised.docx]

**KinetochoreDB: a comprehensive online resource for the kinetochore and its related proteins**

**Supplementary Material**

**Table S1.** GO terms selected in KinetochoreDB using the keyword ‘kinetochore’ from the QuickGO database

| **Aspect** | **GO ID** | **Name** |
| --- | --- | --- |
| Component | GO:0000776 | kinetochore |
| Component | GO:0000777 | condensed chromosome kinetochore |
| Component | GO:0005828 | kinetochore microtubule |
| Component | GO:0000939 | condensed chromosome inner kinetochore |
| Component | GO:0000940 | condensed chromosome outer kinetochore |
| Component | GO:0000778 | condensed nuclear chromosome kinetochore |
| Component | GO:0000941 | condensed nuclear chromosome inner kinetochore |
| Component | GO:0000942 | condensed nuclear chromosome outer kinetochore |
| Component | GO:0031617 | NMS complex |
| Component | GO:0042729 | DASH complex |
| Component | GO:0005818 | aster |
| Component | GO:1990423 | RZZ complex |
| Component | GO:0000817 | COMA complex |
| Component | GO:0031518 | CBF3 complex |
| Component | GO:0031262 | Ndc80 complex |
| Component | GO:0033551 | monopolin complex |
| Component | GO:0044816 | Nsk1-Dlc1 complex |
| Component | GO:1990298 | bub1-bub3 complex |
| Component | GO:0000444 | MIS12/MIND type complex |
| Component | GO:0000818 | nuclear MIS12/MIND complex |
| Component | GO:0005868 | cytoplasmic dynein complex |
| Component | GO:0061638 | CENP-A containing chromatin |
| Component | GO:0032133 | chromosome passenger complex |
| Component | GO:0000779 | condensed chromosome, centromeric region |
| Component | GO:0000780 | condensed nuclear chromosome, centromeric region |
| Function | GO:0043515 | kinetochore binding |
| Function | GO:0003777 | microtubule motor activity |
| Process | GO:0051382 | kinetochore assembly |
| Process | GO:0051383 | kinetochore organization |
| Process | GO:0090234 | regulation of kinetochore assembly |
| Process | GO:0034501 | protein localization to kinetochore |
| Process | GO:1990299 | Bub1-Bub3 complex localization to kinetochore |
| Process | GO:0008608 | attachment of spindle microtubules to kinetochore |
| Process | GO:0072356 | chromosome passenger complex localization to kinetochore |
| Process | GO:0051315 | attachment of mitotic spindle microtubules to kinetochore |
| Process | GO:0051988 | regulation of attachment of spindle microtubules to kinetochore |
| Process | GO:1903394 | protein localization to kinetochore involved in kinetochore assembly |
| Process | GO:0051987 | positive regulation of attachment of spindle microtubules to kinetochore |
| Process | GO:0051316 | attachment of spindle microtubules to kinetochore involved in meiotic chromosome segregation |
| Process | GO:0051455 | attachment of spindle microtubules to kinetochore involved in homologous chromosome segregation |
| Process | GO:0051456 | attachment of spindle microtubules to kinetochore involved in meiotic sister chromatid segregation |
| Process | GO:2000751 | histone H3-T3 phosphorylation involved in chromosome passenger complex localization to kinetochore |
| Process | GO:1902423 | regulation of attachment of spindle microtubules to kinetochore involved in mitotic sister chromatid segregation |
| Process | GO:2000817 | regulation of histone H3-T3 phosphorylation involved in chromosome passenger complex localization to kinetochore |
| Process | GO:1902424 | negative regulation of attachment of spindle microtubules to kinetochore involved in mitotic sister chromatid segregation |
| Process | GO:1902425 | positive regulation of attachment of spindle microtubules to kinetochore involved in mitotic sister chromatid segregation |
| Process | GO:0098653 | centromere clustering |
| Process | GO:0031134 | sister chromatid biorientation |
| Process | GO:2000574 | regulation of microtubule motor activity |
| Process | GO:0072766 | centromere clustering at the nuclear periphery |
| Process | GO:2000575 | negative regulation of microtubule motor activity |
| Process | GO:2000576 | positive regulation of microtubule motor activity |
| Process | GO:0034508 | centromere complex assembly |

A


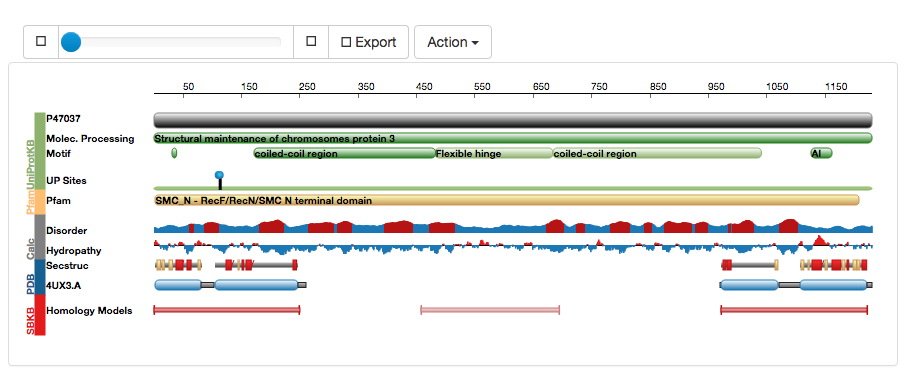


B


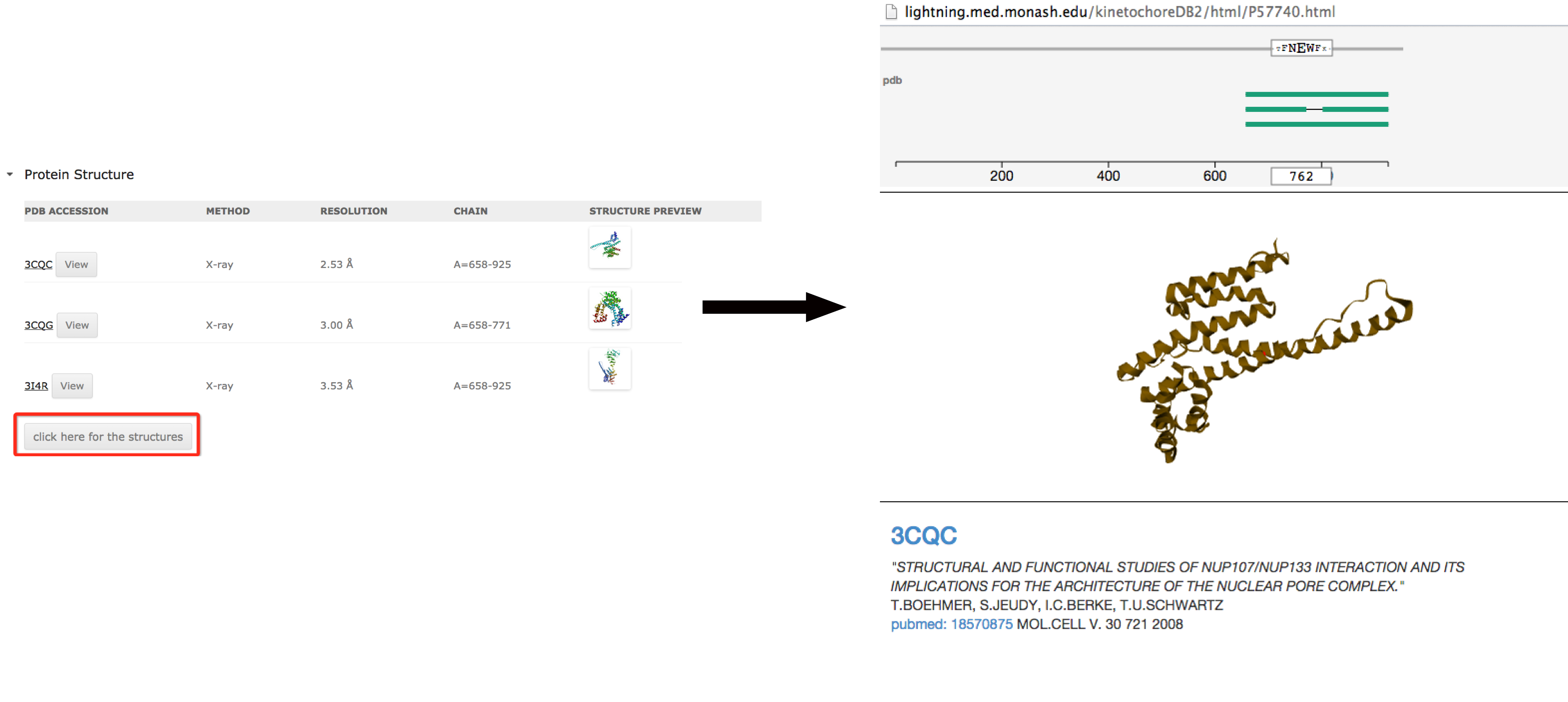


C


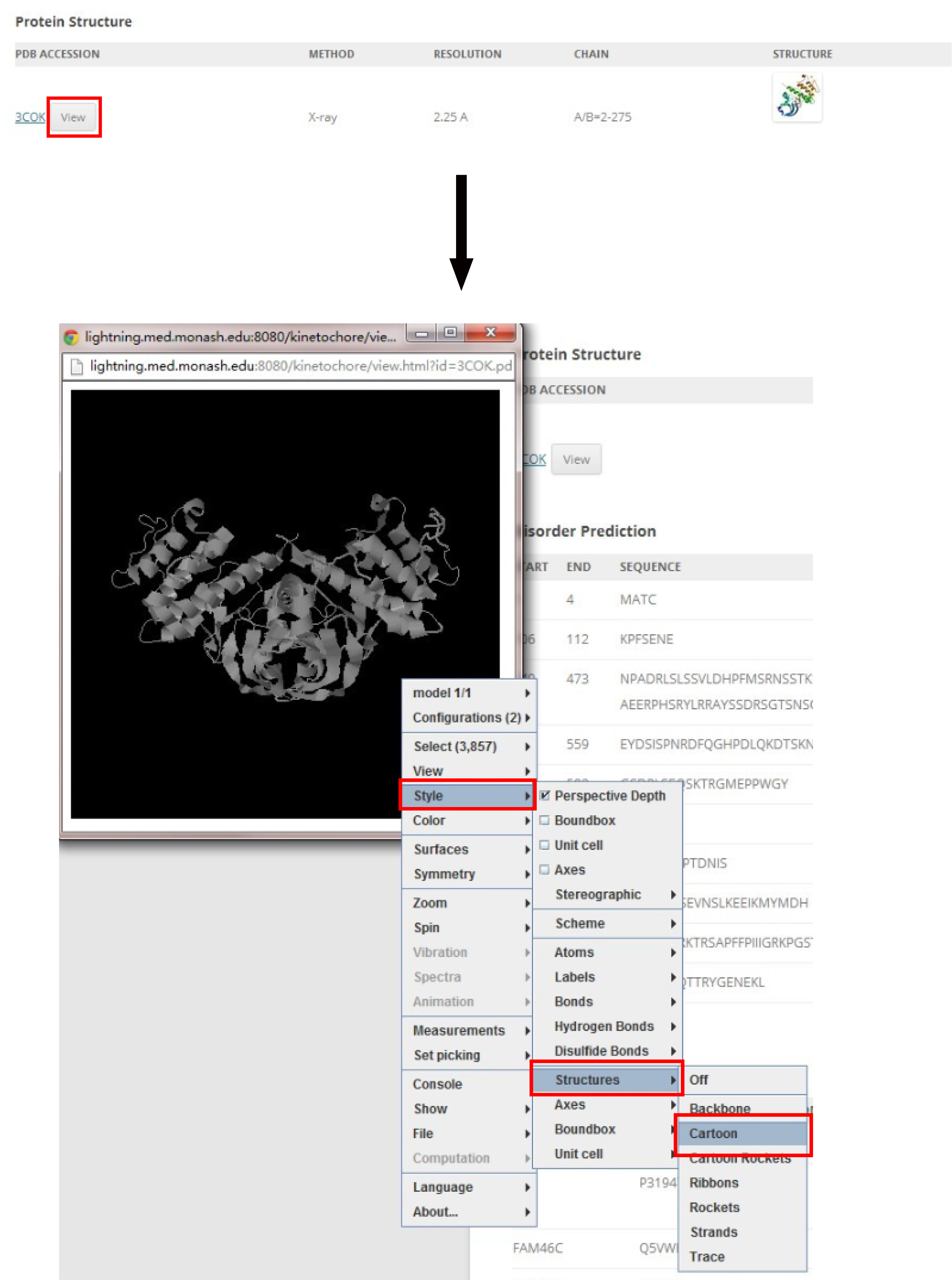


**Figure S1.** JQuery implementation for protein entries in KinetochoreDB. (**A**) Protein overview. (**B**) Protein structure view in an ensemble way with pViz. (**C**) Protein single structure view with Jmol.


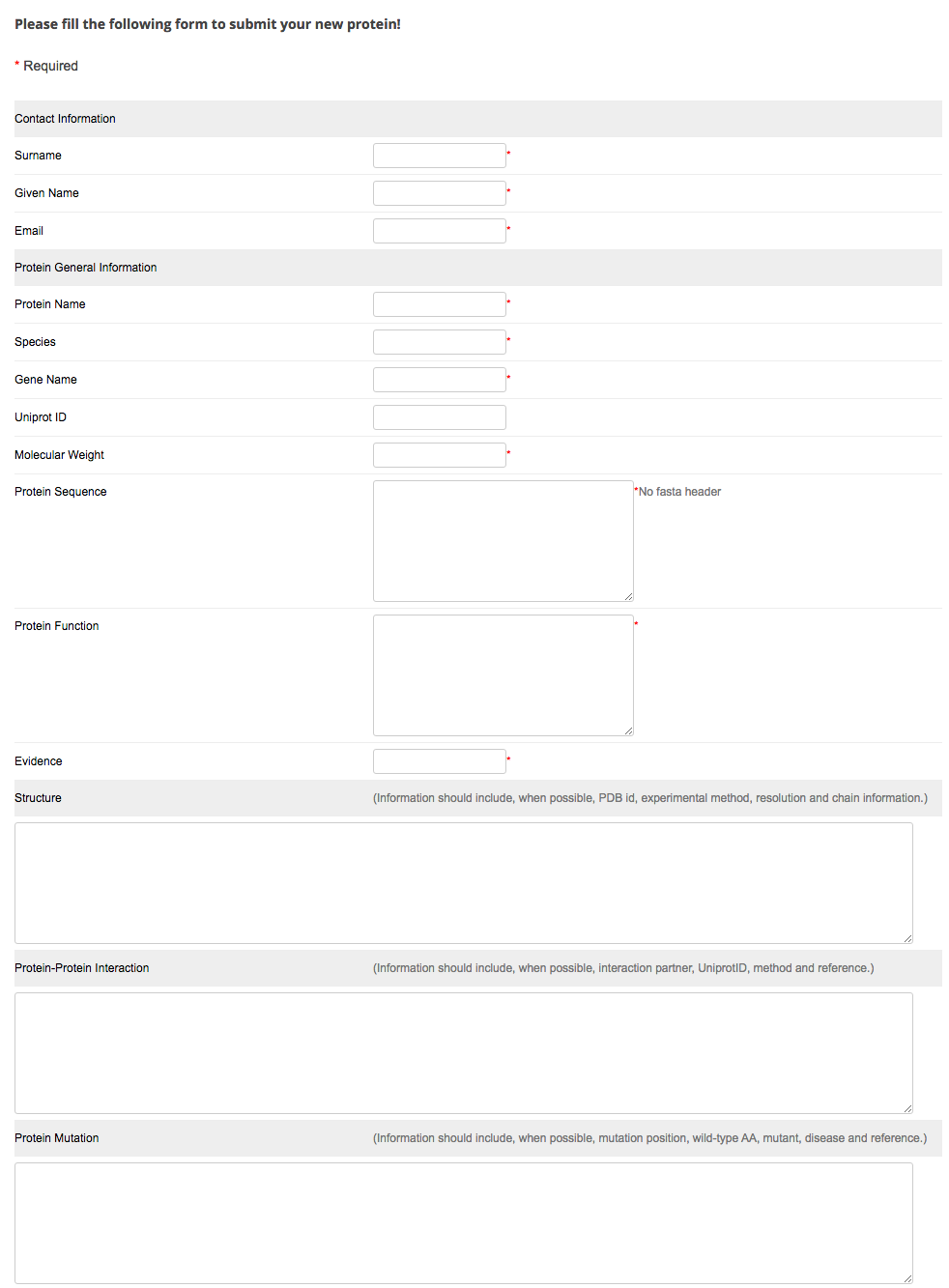


**Figure S2.** Submission page for the users to add a new protein entry.
